# Editorial: Reactive oxygen species signaling and immune diseases

**DOI:** 10.3389/fimmu.2024.1422095

**Published:** 2024-05-21

**Authors:** Saikolappan Sankaralingam, Gopi Kolluru

**Affiliations:** ^1^ Department of Molecular Biology, Amico Dx. LLC, Houston, TX, United States; ^2^ Departments of Pathology Louisiana State University Health Shreveport, Shreveport, LA, United States; ^3^ Department of Molecular and Cellular Physiology, Louisiana State University Health Shreveport, Shreveport, LA, United States; ^4^ Department of Cellular Biology and Anatomy, Louisiana State University Health Shreveport, Shreveport, LA, United States

**Keywords:** ROS, immune diseases, signaling, autoimmune disease, inflammation

Reactive Oxygen Species (ROS) has been extensively studied over the years for its multiple roles associated with the immune system. ROS are central players in the immune cell receptor signaling, and activation, antigen cross-presentation and chemotaxis (1). Increasing evidence indicates that redox balance is an important factor required to maintain a stable immune state and to prevent the development of autoimmune diseases and subsequent tissue damage (2). At moderately elevated levels, ROS may act as a secondary messenger and control various signaling pathways that can activate many transcription factors, including HIF-α, AP-1, ATF, FOXO and NF-κB, which are essential for the maintenance of immunity and inflammatory responses. Thus, ROS can be a dynamic and integral factor for the regulation of signaling networks associated with inflammatory diseases, autoimmune diseases, immunodeficiency diseases, and hypersensitivity.

Although there is no comprehensive data available to depict the morbidity and mortality of immune diseases. Reports indicate nearly 4% to 8% of the world’s population is affected by autoimmune diseases (3), and nearly 1 in 10,000 people are affected by primary immunodeficiency disease. Adding to this burden is the prevalence of hypersensitivity and inflammatory diseases, which highlights the ever-growing need for research to understand the etiology and associated molecular signaling. This current Research Topic is one such effort, to explore the dynamics of ROS signaling emphasizing their impact on therapeutics and the management of immune diseases. In this Research Topic, we have three original articles starting with Berman-Riu et al., presenting the effects of ROS signaling in naïve B cell activation and differentiation to memory B cells observed in Common Variable Immunodeficiency (CVID) patients. Chaumond et al., present their observations on the effect of ROS in the development of the innate immune memory by non-immune cells during Staphylococcus aureus infection, and Sun et al., discuss their observations on how Ferulic acid (FA) supplements inhibit retinal pathological angiogenesis by modulating microglia/macrophage polarization through the ROS/NF-κB axis.

This Research Topic also presents some very interesting and diverse review articles. Manoharan et al., in their review focus on redox factors involved in the activation of immune response and the role of ROS in innate immunity through oxidative modification of proteins. Kannan et al., in their review, revisit the role of ROS signaling in SLE. We also have Teng et al., discuss the relationship between Tumor-associated macrophages (TAMs), Fatty acid oxidation (FAO), and ROS in tumor development and Shu et al., discussing ROS formation and its effect on CD4+ T cell-mediated inflammation. Tang et al., in their review, describe the effects of ROS on ischemia-reperfusion injury and non-alcoholic fatty liver injury through inflammation and cell death. Overall, this Research Topic brings comprehensive research information highlighting ROS signaling mechanisms and their effects as a potential area of focus for application in the development of therapeutic and care plans for immune diseases.

## Author contributions

SS: Writing – review & editing. GK: Writing – review & editing.
